# Supplementary material for: A unified analysis of evolutionary and population constraint in protein domains highlights structural features and pathogenic sites
Source: Commun Biol. 2024 Apr 11;7:447. doi: 10.1038/s42003-024-06117-5 (PMC11009406; doi:10.1038/s42003-024-06117-5)
Supplement: Supplementary file 2 — Supplementary Information [file 42003_2024_6117_MOESM2_ESM.pdf]

A unified analysis of evolutionary and population constraint in protein domains highlights structural features and pathogenic sites

Stuart A. MacGowan[1], Fábio Madeira[1,2], Thiago Britto-Borges[1,3] and Geoffrey J. Barton[1*]

1. Division of Computational Biology
   School of Life Sciences
   University of Dundee
   Dow Street
   Dundee, DD1 5EH
   Scotland, UK.
2. Present address – European Bioinformatics Institute (EMBL-EBI), Wellcome Trust Genome Campus, Hinxton, Cambridge, CB10 1SD, UK.
3. Present address – Section of Bioinformatics and Systems Cardiology, Department of Internal Medicine III and Klaus Tschira Institute for Integrative Computational Cardiology, Heidelberg University Hospital, Heidelberg, Germany.

Contents

Supplementary material 2

Supplementary tables 2

Supplementary figures 4

Supplementary material

Supplementary tables

Supplementary Table 1. The association of missense enrichment score category and solvent exposure class. Contingency table of frequencies and standardised residuals. $\chi^2 = 1285$, df = 4, p ≈ 0, n = 105,385. The data represent 105,385 sites within 592 Pfam domains, covering 2,603,483 residues in human proteins.

	Core	Partially exposed	Surface
		Observed Frequencies	
Depleted	1,569	1,138	1,287
Neutral	22,917	21,679	52,950
Enriched	432	726	2,687
		Standardised Residuals	
Depleted	23	9.5	-28
Neutral	-4	-3	6
Enriched	-18	-5	20

Supplementary Table 2. The classification of Pfam domains by population missense constraint and evolutionary divergence.

	Families[1]	Sites	Residues (human)	Missense (gnomAD)
CMD	557	3,156	268,609	73,231
CMN	605	53,025	969,480	424,062
CME	479	1,695	68,989	51,667
UMD	419	1,017	48,531	13,188
UMN	605	48,541	1,213,464	574,939
UME	548	2,356	213,231	130,620

Table footnotes: 1. Excludes protein families that did not have at least one missense depleted column (see Methods). Abbreviations: CMD = conserved and missense depleted; CME = conserved and missense enriched; CMN = conserved and missense neutral; UME = unconserved and missense enriched; UMD = unconserved and missense depleted; UMN = unconserved and missense neutral.

Supplementary Table 3. The enrichment of functional features in UMD vs. UME positions in Pfam domains stratified by RSA.

Feature	RSA	Odds ratio	p	95% CI
Protein-ligand	All	1.59	6.41×10^{-16}	1.42 – 1.77
	Core	0.94	0.78	0.64 – 1.38
	Part Exposed	2.20	2.12×10^{-12}	1.75 – 2.79
	Surface	1.37	0.0001	1.17 – 1.60
Protein-protein	All	1.14	4.54×10^{-11}	1.09 – 1.18
	Core	1.14	0.19	0.94 – 1.39
	Part Exposed	1.27	7.25×10^{-8}	1.16 – 1.39
	Surface	1.50	7.90×10^{-65}	1.44 – 1.57
Pathogenic	All	6.41	4.98×10^{-47}	5.08 – 8.08
	Core	5.05	0.00009	2.06 – 13.58
	Part Exposed	4.90	3.46×10^{-10}	2.88 – 8.55
	Surface	6.48	1.48×10^{-27}	4.79 – 8.69

Supplementary figures

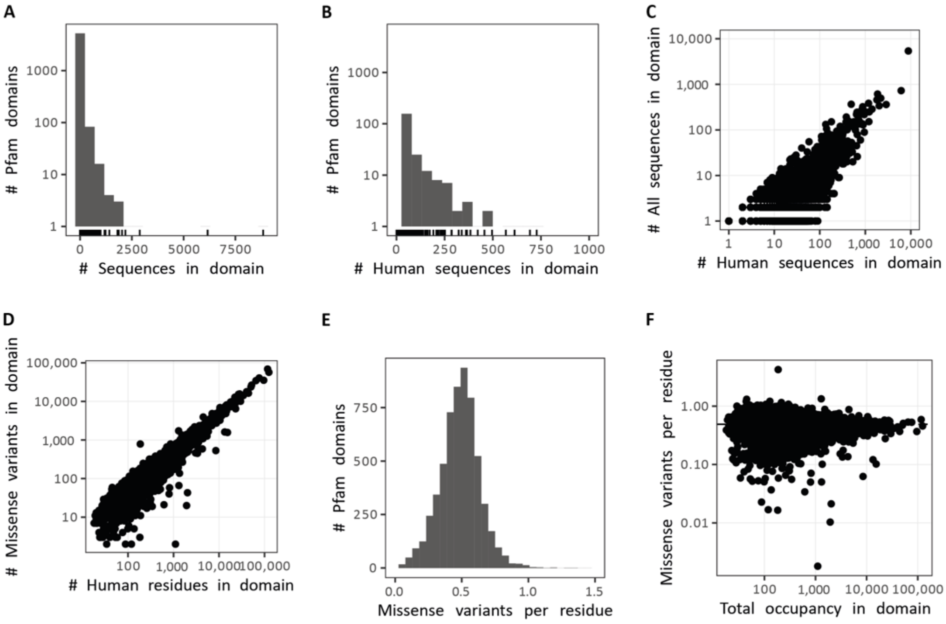

Supplementary Figure 1. Additional plots showing the distribution of human sequences and missense variants in our Pfam-gnomAD dataset. A. Number of sequences (all species) in Pfams with at least one human sequence. B. Number of human sequences in these Pfam domains. C. Number of human sequences vs. total number of sequences. D. Number of missense variants vs. number of human sequences in Pfam domains. E. Histogram of the ratio of missense variants to human residues. F. The missense residue ratio vs. the number of human residues in the domain.

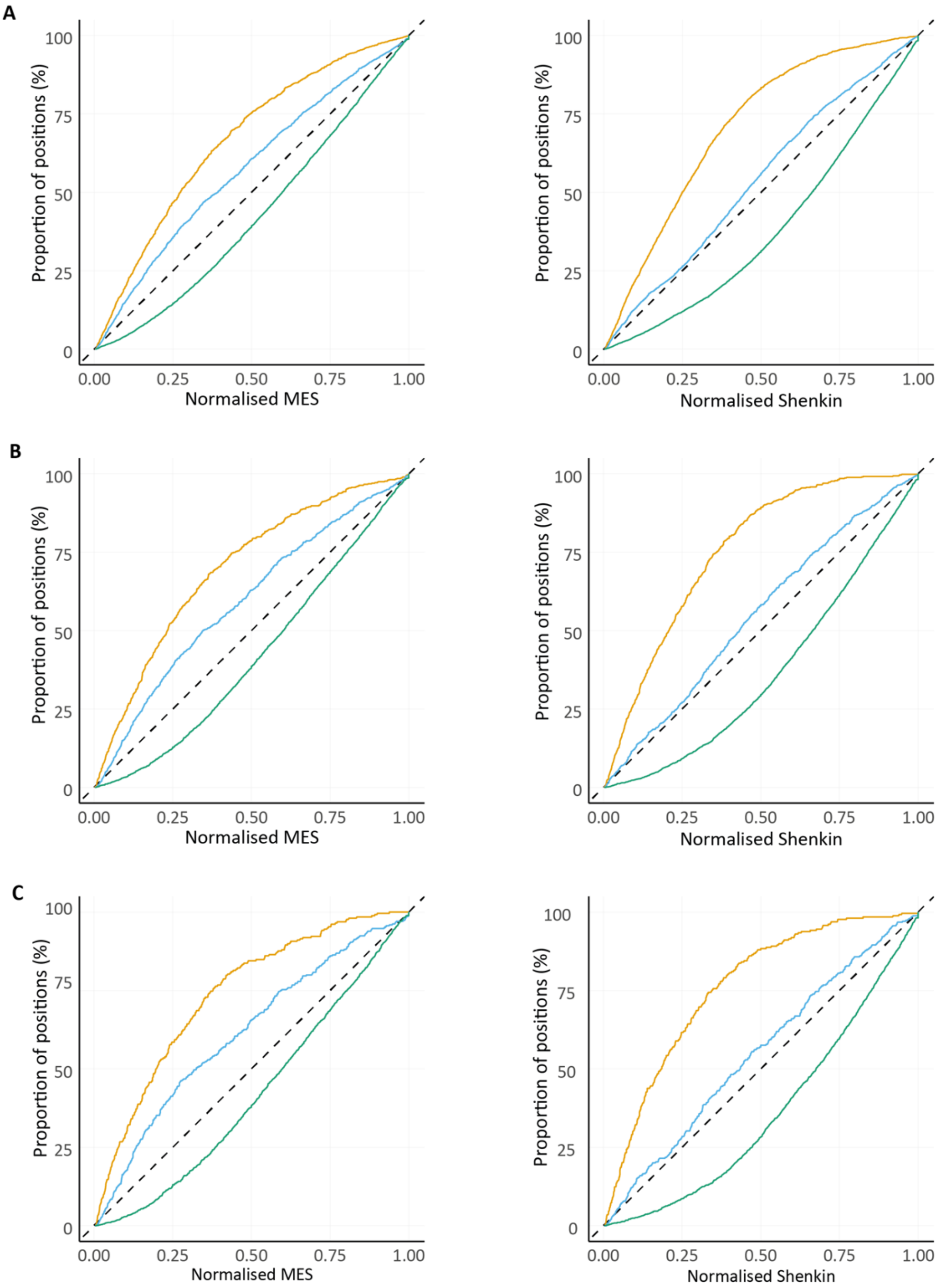

Supplementary Figure 2. Association between relative solvent accessibility class and the normalised missense enrichment score (left panels) or normalised Shenkin divergence (right panels) at increasing alignment depths (A ≥ 50, B ≥ 100 and C ≥ 200 human sequences in the Pfam domain). Each line shows the cumulative distributions of normalised MES or normalised Shenkin for Pfam domain positions over the different RSA classes (Orange = Core, blue = Partially Exposed and green = Surface). For example, from panel A we can see that < 50% of buried positions are in the 25% least missense variable residues amongst Pfam domains with at least 50 human sequences whilst panel C shows that > 50 % of buried positions are in the 25% least missense variable residues amongst Pfam domains with at least 200 human sequences, indicating that MES is more strongly associated with RSA class in deeper alignments.

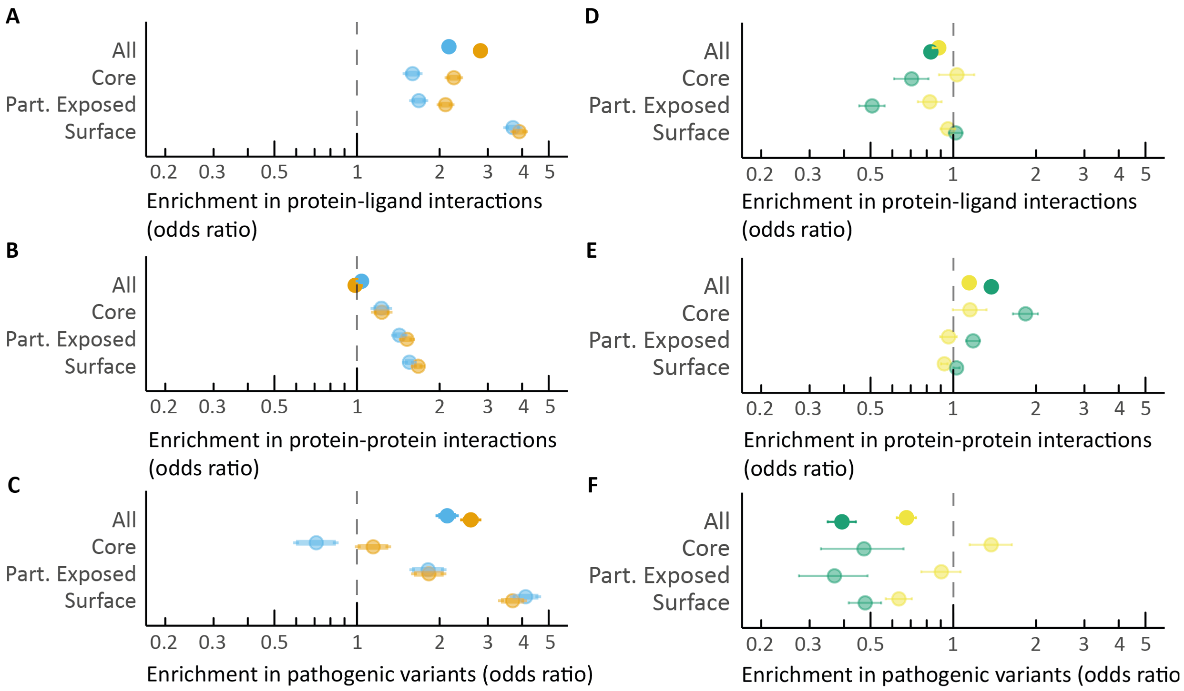

Supplementary Figure 3: The structural and functional properties of A-C. missense depleted sites (blue) compared to evolutionarily conserved (orange) sites and D-F. missense enriched sites (yellow) compared to evolutionarily divergent sites (green), all stratified by solvent exposure class. Odds ratios (95% CI) measuring the enrichment of A, D. protein-ligand interactions, B, E. protein-protein interactions and C, F. pathogenic variants.

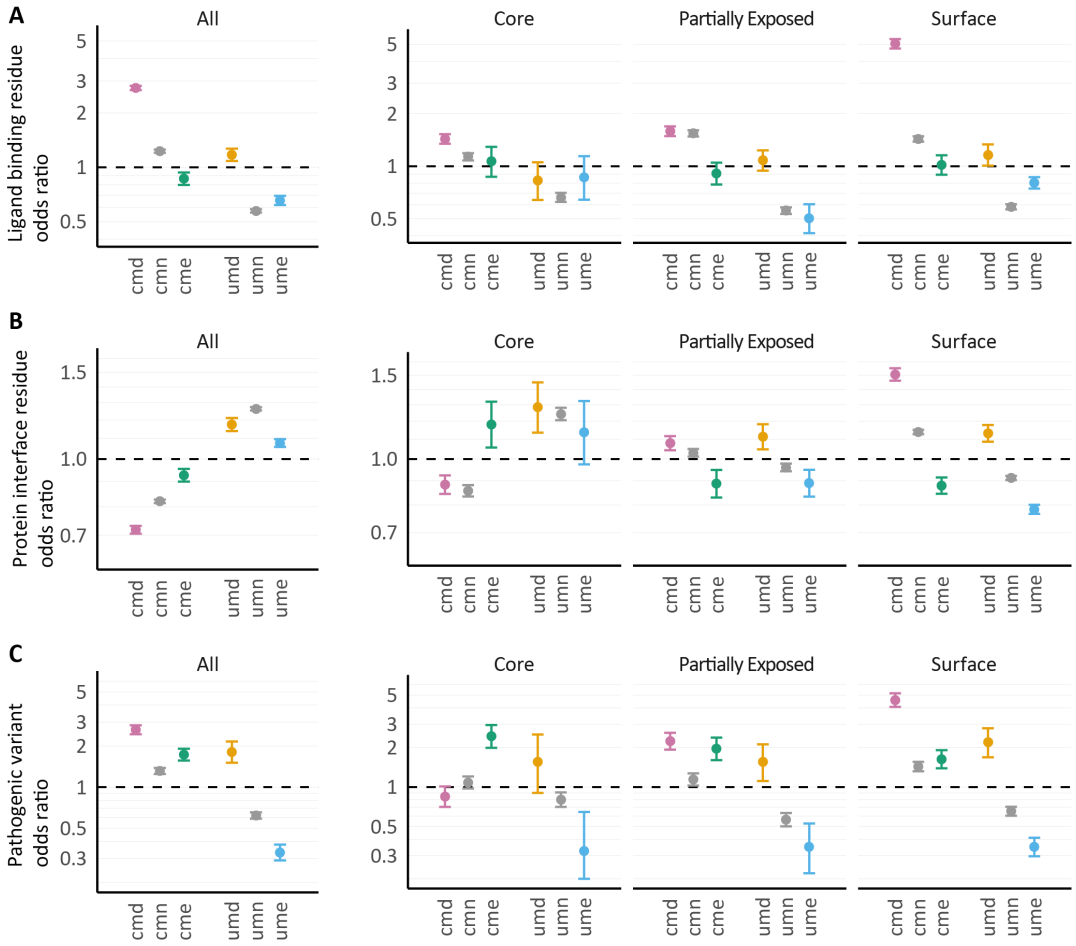

*Supplementary Figure 4. The enrichment of interactions and clinical variants in different regions of the conservation plane stratified by relative solvent accessibility (RSA). Panes **A-C**: All=All RSA classes; Core=Buried sites; Surface=Exposed sites; Partially Exposed=Intermediary RSA. See Methods for definitions. **A.** Odds ratios of the enrichment of protein-ligand interacting points) and unconserved positions (second group of three points) by missense depletion, missense neutrality and missense enrichment. Error bars indicate 95 % CI. N = 1,849,014 residues. **B.** Protein-Protein Interaction sites (PPIs) N = 2,355,592 residues. **C.** ClinVar Pathogenic site enrichments relative to the gnomAD missense background. Supplementary Table 2 presents related data focussed on comparing UMD and UME positions. N = 840,894 variants.*

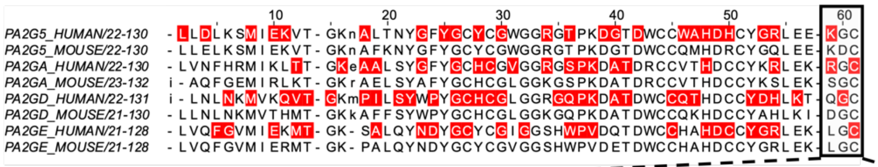

Pfam Stockholm alignment file			
Column	UniProt Name	UniProt ID	Residue
59	PA2G5_HUMAN	P39877	77
59	PA2GA_HUMAN	P14555	77
61	-	-	79
60	PA2GD_HUMAN	Q9UNK4	78
61	PA2GE_HUMAN	Q9NZK7	77

VEP Annotated gnomAD VCF					
Row:CHROM	Row:POS	Row:REF	Row:Alt	VEP:SwissProt	VEP:HGVSp
1	20416327	G	C	P39877	p.Lys77Asn
1	20304570	C	G	P14555	p.Arg77Pro
1	20304563	A	C	-	p.Cys79Trp
1	20442060	C	G	Q9UNK4	p.Gly78Arg
1	20248847	C	T	Q9NZK7	p.Cys77Tyr

VarAlign (Biopython.AlignIO)

VarAlign (PyVCF, Pandas)

Supplementary Figure 5. Schematic illustrating how missense variants from gnomAD are mapped to Pfam domain multiple sequence alignments. An excerpt of a Pfam domain family alignment is shown where sites with missense variants in gnomAD are highlighted red. The tables below the alignments show the UniProt residue mappings that are extracted from the Pfam Stockholm alignment files and variant records from the gnomAD VCF with Ensembl VEP annotations. The merge keys between the tables are indicated by the dashed lines.
