# Supplementary material for: A unified analysis of evolutionary and population constraint in protein domains highlights structural features and pathogenic sites
Source: Commun Biol. 2024 Apr 11;7:447. doi: 10.1038/s42003-024-06117-5 (PMC11009406; doi:10.1038/s42003-024-06117-5)
Supplement: Supplementary file 3 — Description of Additional Supplementary Files [file 42003_2024_6117_MOESM3_ESM.pdf]

Description of Additional Supplementary Files

File name: Supplementary Data 1

Description: Strongly coupled sites table downloaded from the EVcouplings webserver after running the STF-1 nuclear receptor ligand binding domain sequence obtained from the Pfam domain alignment (PF00104).
